# The Saltpan Microbiome Is Structured by Sediment Depth and Minimally Influenced by Variable Hydration

**DOI:** 10.3390/microorganisms8040538

**Published:** 2020-04-08

**Authors:** Eric A. Weingarten, Lauren A. Lawson, Colin R. Jackson

**Affiliations:** Department of Biology, University of Mississippi, University, MS 38677, USA; lalawson@go.olemiss.edu (L.A.L.); cjackson@olemiss.edu (C.R.J.)

**Keywords:** tidal wetlands, soil microbial communities, 16S rRNA, halophiles

## Abstract

Saltpans are a class of ephemeral wetland characterized by alternating periods of inundation, rising salinity, and desiccation. We obtained soil cores from a saltpan on the Mississippi Gulf coast in both the inundated and desiccated state. The microbiomes of surface and 30 cm deep sediment were determined using Illumina sequencing of the V4 region of the 16S rRNA gene. Bacterial and archaeal community composition differed significantly between sediment depths but did not differ between inundated and desiccated states. Well-represented taxa included marine microorganisms as well as multiple halophiles, both observed in greater proportions in surface sediment. Functional inference of metagenomic data showed that saltpan sediments in the inundated state had greater potential for microbial activity and that several energetic and degradation pathways were more prevalent in saltpan sediment than in nearby tidal marsh sediment. Microbial communities within saltpan sediments differed in composition from those in adjacent freshwater and brackish marshes. These findings indicate that the bacterial and archaeal microbiomes of saltpans are highly stratified by sediment depth and are only minimally influenced by changes in hydration. The surface sediment community is likely isolated from the shallow subsurface community by compaction, with the microbial community dominated by marine and terrestrial halophiles.

## 1. Introduction

Saltpans are a class of hypersaline wetland that are often intermixed with tidal freshwater, brackish, and saltwater marshes [1,2]. Saltpans are characterized by their unique hydrology, with alternation between ephemeral saturation during storm tides, salinification, and desiccation when surface water evaporates [3]. Saltpan formation can occur in high marsh plateaus and within shallow depressions of tidal marshes, where salts can become trapped and concentrate when water evaporates [2]. Saltpans are related to the more general class of tidal flats [4,5], but are distinguished by their periodically hypersaline conditions and sparse vegetation coverage consisting of only emergent, very salt-tolerant species [6].

As saltwater intrusion progresses into tidal wetlands, it is projected that high salt marsh and salt flat wetland types will replace areas historically occupied by tidal freshwater, brackish, and salt marshes [2,7]. Each of these wetland types is conducive to the formation of saltpans. Despite projections of saltpan area expansion, very few studies have yet examined the microbial communities within tidal flats [4,8,9] and none, to our knowledge, have specifically characterized the bacterial community of saltpan sediments. Microbial mediation of energy flows and nutrient cycles within wetlands has been well described [10,11,12], but it is, as yet, unclear whether microbial communities and processes in tidal flats and saltpans resemble those in vegetated, tidal wetlands.

It was hypothesized that the structure of the saltpan microbiome would be strongly dependent on hydration state. Previous studies of wetland microbiomes have found that the duration of flood events in ephemeral wetlands is the most significant variable in soil community composition, exceeding the effects of soil physico-chemistry or depth [13,14]. The effect of soil hydration on community structure is well studied [15,16,17] and differences in soil moisture can be particularly strong determinants of the rare fraction of the microbial community [18]. It was predicted that the abundant fraction of the saltpan microbiome would shift in response to flooding, from a halophile and desiccation-tolerant community in the dry state to a community dominated by transient marine species in the flooded state. It was also expected that these shifts would be more pronounced in the surface sediment than at root zone depth, due to the necessity for water percolation to disturb the subsurface. Hydrologic pulsing may have a strong influence on bacterial metabolic processes, even independent of compositional changes [19] and we hypothesized that inferred metagenomes would show overall greater activities in the hydrated than in the desiccated state.

Here, we characterize the bacterial and archaeal microbiome of sediments in a saltpan located on the Gulf Coast of Mississippi, USA. Sediment cores were collected in the summer while the site was in the desiccated state and again in the fall following a tropical storm generated tide. Communities were characterized using 16S rRNA gene sequencing and compared to sequences recovered from freshwater and brackish marshes located within the same area. Metagenomes of the saltpan samples were inferred using 16S rRNA sequences to estimate potential functional profiles of saltpan sediments. Our results demonstrate that: (1) saltpan microbial communities are strongly clustered by depth within sediment, (2) community composition is minimally affected by hydration level, and (3) saltpan sediments differ significantly from sediments in fresh and brackish marsh both in taxonomic makeup and inferred metagenomes, even though these systems may be immediately adjacent to each other. These findings were in contrast to our hypotheses, emphasizing the unique phyico-chemical and hydrological characteristics of saltpans and how they influence microbiome structure.

## 2. Materials and Methods 

### 2.1. Sample Collection

**Figure 1:**
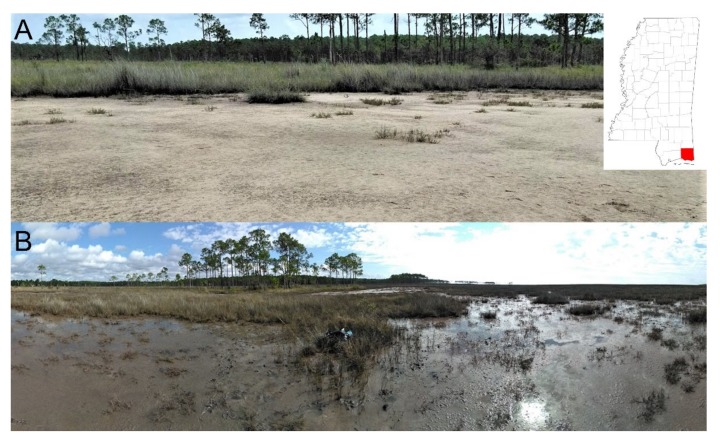
Saltpan wetland within the Grand Bay National Estuarine Research Reserve (GBNERR) on the Mississippi Gulf coast in June 2018 (**A**) under the desiccated state and in October 2018 (**B**) following a storm tide. The storm tide was generated by Tropical Storm Gordon, which made landfall east of Pascagoula, Mississippi on September 5, 2018.

Samples were collected from a saltpan located in Grand Bay National Estuarine Research Reserve (GBNERR, 30°24’24.84”N, 88°24’27.38”W) along the Mississippi Gulf Coast in Jackson County, Mississippi, USA (Figure 1). 

The saltpan wetland type comprises a total of ~106 ha at GBNERR, approximately 1% of the total reserve area [6]. Freshwater, intermediate, tidal salt, and high marsh, by comparison, cover ~3045 ha, or ~42% of the total reserve area. The saltpans are patchily distributed throughout GBNERR, interspersed with salt and intermediate tidal marsh types. Saltpans and higher sand flats at the site are only flooded in spring or following storm tides and can exhibit salinity levels twice that of seawater during the dry phase. Samples were collected on June 18 (dry conditions) and October 3 (flooded conditions), 2018. The flooded conditions followed an inundation event that was a result of storm tides following Tropical Storm Gordon, that made landfall east of Pascagoula, Mississippi, USA, on September 5, 2018. Floodwaters resulted from both >1 m storm surge and >25 cm of local rainfall [20]. At the time of the October sampling, the sediment was saturated but in an ebb state. On each collection date, samples were collected as sediment cores using a sterilized (70% ethanol) 38 cm × 2 cm soil probe. Sediment cores were divided into surface and lowest subsections (30 cm deep). Two cores were taken on each sample date, giving a total of eight samples (2 dates × 2 depths × 2 cores) from the saltpan site. Additional sediment samples were taken from an adjacent fresh and intermediate tidal marsh at GBNERR on June 18, 2018, using the same sampling procedure, with the exception that five cores were taken from each site. Samples were stored on ice until return to the laboratory. Samples were stored frozen until November 2018, when a single 0.25 g subsample of each core was taken for DNA extraction, and the remaining material was used for physico-chemical analyses.

### 2.2. Soil Physico-Chemical Analysis

Saltpan sediment samples were dried at 55 °C for 48 h and then homogenized with a mortar and pestle to crush any aggregates. Electrical conductivity determination was adapted from He et al. 2013 [21]. A 1:5 dried sediment to sterile reverse osmosis (RO) water suspension was shaken on a vortex adapter for 10 min, and then shaken for 8 h at 25 °C on an incubator shaker at 150 rev min^−1^. Samples were centrifuged at 1000× *g* for 5 min. The electrical conductivity of the supernatant was measured using a YSI probe (YSI International, Yellow Spings, OH). Electrical conductivity of the dry sample was estimated by the equation

*E_ec_ = (E_d_ ‒ E_w_)f* (1)

where *E_ec_* is the electrical conductivity of the dry sample, *E_d_* is the conductivity of the dilution, *E_w_* is the conductivity of the R.O. water (0) and *f* is the dilution factor (5) [22]. Final electrical conductivity is presented as practical saline units (PSU). The pH of the supernatant was measured using an accumet AB15 pH electrode (Thermo Fisher Scientific, Waltham, MA). Soil organic matter (OM%) was determined by loss-on-ignition of a dried sample, following ashing at 500 °C for 2 h. Particle size determination was adapted from Kettler et al. 2001 [23]. Dried sediment samples were resuspended in a 1:5 R.O. solution and vortexed for 10 min. The slurry was sieved through a 0.053 mm mesh (no. 270) sieve to capture the sand fraction. The silt/clay suspension was vortexed for 10 min and allowed to settle at room temperature for 4 h. The suspended clay fraction was then poured off from the settled silt fraction. Both the sand and silt fractions were dried at 55°C for 48 h. The final sand/silt/clay percentages were calculated as:

Sand % = (dry sand mass / original dry sample mass) × 100%(2)

Silt % = (dry silt mass / original dry sample mass) × 100%(3)

Clay % = 100 ‒ (Sand % + Silt %)(4)

Electrical conductivity, pH, and organic matter determination were performed in triplicate. The triplicate samples were combined, resuspended, and particle size was determined from the pooled samples.

### 2.3. DNA Extraction, 16S rRNA Gene Amplification, and Sequencing

DNA was extracted from the eight sediment cores using a Qiagen DNeasy PowerSoil DNA Isolation Kit (Qiagen, Germantown, MD, USA), following the standard protocol with the addition of a 10 min incubation at 70 °C prior to bead-beating. Total recovered DNA was amplified targeting the V4 region of the 16S rRNA gene using dual-indexed barcoding and the primers and procedures of Kozich et al. [24]. Then, 1 µL of genomic DNA was combined with 1 µl of each barcoded primer and 17 µL of AccuPrime *Pfx* SuperMix (Life Technologies Corporation, Carlsbad, CA, USA). PCR amplification consisted of a 95 °C hot start for 2 min, followed by 30 cycles of 95 °C (20 s), 55 °C (15 s), 72 °C (2 min), and final elongation at 72 °C for 10 min [24,25]. Successful DNA amplification was confirmed using gel electrophoresis. Amplicon concentration was normalized using a SequalPrep Normalization Plate Kit (Invitrogen Corporation, Carlsbad, CA, USA), and the amplified 16S rRNA gene fragments were sequenced using 251×251 PE reads on the Illumina MiSeq platform at the Molecular and Genomics Core Facility of the University of Mississippi Medical Center (Jackson, MS, USA).

### 2.4. Data Analysis

Illumina sequence data (FASTQ files) were processed using mothur [26] following the pipeline suggested by Schloss et al. [27] and Kozich et al. [24] Sequences were aligned against the Silva database release 132 [28] and classified against version 16 of the Ribosomal Database Project (RDP) database [29]. Sequences attributed to chloroplasts, mitochondria, Eukarya, or which were unclassified at the kingdom level were removed, as were sequences that were potential chimeras. Archaeal sequences were retained in the saltpan samples as it was expected those taxa would be major constituents of the hypersaline conditions. Saltpan sequences were analyzed in parallel with archaeal and bacterial sequences run through the same pipeline but curated differently. Microbial sequences from all samples were grouped into operational taxonomic units (OTUs), based on 97% similarity. As there was a ~10:1 proportion of bacterial to archaeal sequences, the bacterial dataset had any OTUs representing < 0.01% of the data removed [25] while all archaeal data was retained. Saltpan bacterial sequence data was rarefied to 731 sequences and archaeal data to 57 sequences. 

Phylum and genus level differences in taxonomy between hydration states and sediment depth were determined statistically using MANOVA. A Bray–Curtis dissimilarity matrix, based on sequence relative abundance, was used to calculate pairwise compositional differences between the hydration states and depth at the saltpan site. Separate matrices were calculated for bacterial and archaeal datasets, to determine whether those domains responded differently to hydration or were vertically structured. A Spearman correlation was performed, comparing the OTU abundance table to the distance matrix, to determine which taxa were most influential in separating environmental samples. This was calculated using the corr.axes function in mothur. Bray–Curtis matrices were also established to compare the saltpan sediment microbiome to that of the freshwater and intermediate salinity sites. For all dissimilarity matrices, non-metric multidimensional scaling (NMDS) ordination was used to visually represent differences in microbial community composition. NMDS ordinations were established using the metaMDS function in the Vegan package [30] in R version 3.6.1. PERMANOVA was used to compute statistical differences in composition between sites/hydrations/depth based on a dissimilarity matrix. The Metastats [31] command in mothur was used to compare the composition of the different wetland samples and identify members that statistically distinguish, or are most unique, to each group. Alpha diversity was assessed using observed species richness (Sobs; an estimation of the number of OTUs in a sample when standardized to the same number of sequences), as the measure for species richness and the inverse Simpson metric as the measure for diversity with species evenness was also taken into account. A two-way ANOVA was used to quantify differences in alpha diversity by the hydration state and the sediment depth.

The Piphillin metagenomic inference tool [32] was used to predict metagenomic pathways that might be differentially present between the hydration states of the saltpan, the sediment layers, and/or between the different wetland classes based on the taxonomic differences observed. Piphillin takes an OTU abundance table and metadata file and produces a similar samplewise abundance table of predicted gene abundances. BioCyc release 22.5 [33] was used as the reference database against a 97% identity cutoff. Following the methods suggested by Poret-Peterson et al. [34], features with <100 expressed genes in each sample were eliminated from the count table produced by Piphillin. This filter was not applied to the BioCyc abundance table produced for the three wetland types combined, as many genes were only present at one of the three sites, reflecting the strong differences observed between those sites. The DESeq2 package [35] was used to calculate log_2_ fold changes and adjusted *p*-values for each feature across each of the treatments. Computed log_2_FC and *p*-values were uploaded to the MetaCyc Pathway Tools Omics Dashboard [33], using a 0-centered scale which groups BioCyc features into different cellular functions. The degradation pathways for cellulose, chitin, and starch, the energetic pathways for aerobic respiration, methanogenesis, CO_2_ fixation, N-fixation, anammox, nitrate reduction I, dissimilatory sulfate reduction I, and fermentation to alcohols, short-chain fatty acids, and of pyruvate, as well as assimilatory pathways for phosphorus, were selected for analysis. Log_2_FC changes were used to infer the magnitude of subsystem differences between sites/conditions. Enrichment analysis, which calculates -log_10_ (*p*-values) based on the number of differentially expressed features within each subsystem was used as a measure of significance. For downstream analysis, the importance of log_2_FC was emphasized over -log_10_ (*p*-value), as each of our selected subsystems are at a high-level functional group classification, with large differences in the number of component pathways within each subsystem. Thus, those subsystems with a large number of daughter pathways, such as aerobic respiration and CO_2_ fixation, tended to yield significant values, while pathways with fewer components tended to show much larger fold changes but were not significant. Mantel’s tests were performed to show the correlations between taxonomic and metagenomic distance matrices [36]. Strong and significant correlations increase the confidence that inferred genomes reflect differences in the taxonomic data. 

## 3. Results

In terms of physico-chemistry, saltpan sediments in the desiccated surface exhibited the highest salinity of any depth/hydration combination (Equation 1, Table 1). Saltpan sediments in the hydrated surface, as well as in the hydrated and desiccated subsurface, showed intermediate salinity comparable to a typical brackish marsh. Surface sediments had a higher proportion of sand relative to silt, while the opposite was true for subsurface sediments (Equations 2–4). Organic matter content and slightly acidic pH were similar across all observed conditions. 

**Figure 2:**
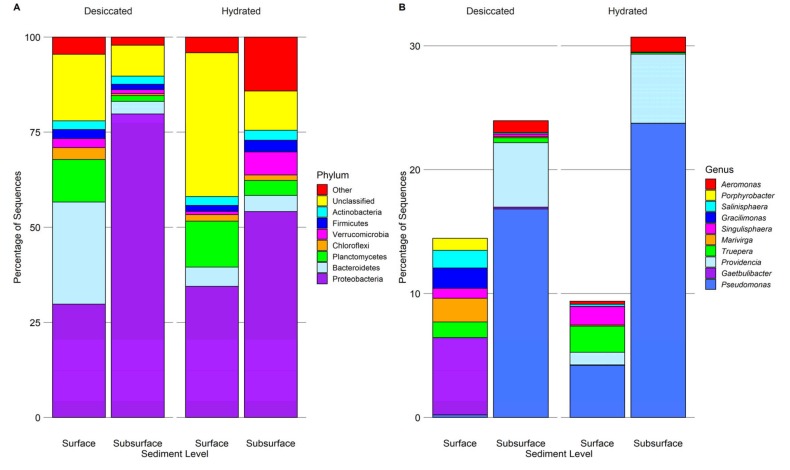
Representation of bacterial phyla (**A**) and genera (**B**) in saltpan wetland sediments collected from the Mississippi Gulf Coast and characterized by 16S rRNA gene sequencing. Major phyla (**A**) are shown as proportions of the total dataset of 96,391 bacterial sequences. Minor phyla are grouped as “other.” The ten most abundant overall genera (**B**) are shown as proportions of the total dataset. These ten represent 19.6% of the total sequences and ~82% of the sequences identified at the genus level.

The 16S rRNA sequence dataset contained 96,391 bacterial sequences with counts per sample ranging between 731 and 37,414, with an average of 12,049 sequences. There were 10,440 archaeal sequences, with counts per sample ranging from 57 to 6498 sequences, with an average of 1305 sequences. Average subsample coverage was 81% for Archaea and 84% for Bacteria. Moreover, 79% of the bacterial sequences could be identified at the phylum level and seven bacterial phyla accounted for >95% of those classified sequences: Proteobacteria (49.5% of all sequences), Bacteroidetes (9.9%), Planctomycetes (7.2%), Verrucomicrobia (2.6%), Actinobacteria (2.3%), Firmicutes (2.1%), and Chloroflexi (1.7%) (Figure 2A). 

24% of the bacterial sequences could be identified to the genus level (Figure 2B). Of those, *Pseudomonas* (11.3%), *Providencia* (3.0%), *Gaetbulibacter* (1.6%), and *Truepera* (1.0%) made up the largest proportion. None of the major bacterial phyla or genera differed significantly in relative abundance between either soil depth or hydration state (*p* > 0.05, MANOVA). However, the desiccated surface condition was significantly higher in *Gaetbulibacter, Marivirga, Gracilimonas,* and *Marivita* abundance compared to all other conditions (*p* <0.05, MANOVA). Unlike the other three conditions, *Pseudomonas* made up a relatively low proportion of the desiccated surface sediment, although this pairwise comparison was non-significant (*p* = 0.35). Phylum Euryarchaeota accounted for 90% of the archaeal sequences with Thaumarchaeota (2.8%) and Pacearchaeota (2.0%) making up a minor portion of the sequences. Just 12% of the archaeal sequences were identified to the genus level, with *Halarchaeum* making up 89% of those sequences.

Bacterial sequences clustered into 3352 OTUs, of which 1463 were represented by only a single sequence. Among the highest represented OTUs in the dataset, 14 contained >1000 sequences and together accounted for almost 45% of all sequences. The first, second, and seventh most abundant OTUs were each classified to the family Enterobacteriaceae, while other predominant bacterial OTUs were members of the Pseudomonadaceae, Flavobacteriaceae, Rhodobacteraceae, Rhodothermaceae, and Sphingomonadaceae. Both bacterial species richness (*S*_obs_) and diversity (Inverse Simpson) were similar between the surface and subsurface layers, and neither showed significant differences based on hydration state (*p* = 0.47–0.93; ANOVA). Average species richness was 254.6 ± 5.1 for desiccated surface sediments, 116.6 ± 24.4 for desiccated subsurface sediments, 198.5 ± 21.7 for hydrated surface sediments, and 218.3 ± 145.7 for hydrated subsurface sediments. Average inverse Simpson diversity was 58.9 ± 31.8 for desiccated surface sediments, 7.8 ± 2.4 for desiccated subsurface sediments, 21.6 ± 2.8 for hydrated surface sediments, and 80.6 ± 75.1 for hydrated subsurface sediments.

**Figure 3:**
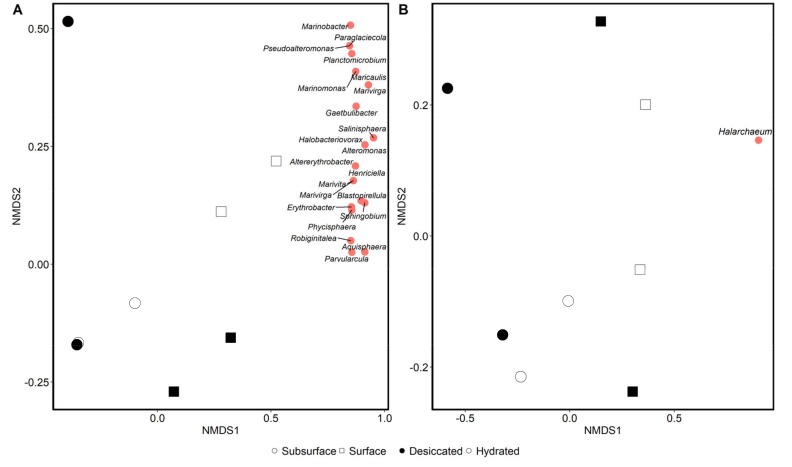
Non-metric multidimensional scaling (NMDS) ordination of bacterial (**A**) and archaeal (**B**) communities in saltpan wetland sediments collected from the Mississippi Gulf Coast. Sediment was collected from the surface and the 30 cm subsurface. Duplicate cores were taken from the same site in a desiccated state and flooded state. Ordinations are based on Bray–Curtis dissimilarity matrices derived from 16S rRNA gene sequence data. Red circles indicate genera that were most influential in separating samples and the position in the NMDS ordination that they pulled samples toward. Only one operational taxonomic unit (OTU) (*Halarchaeum*) was influential in separating archaeal communities.

A Bray–Curtis dissimilarity matrix separated both archaeal and bacterial communities according to soil depth (Figure 3). Bacterial communities differed significantly in their composition according to soil depth (*p* = 0.041; PERMANOVA), while the effect of hydration was non-significant (*p* = 0.585). The same pattern in community structure was observed for the archaeal sequences, with the effect of depth being on the edge of significance (*p* = 0.054) and the effect of hydration being non-significant (*p* = 0.498). Spearman correlation showed 22 bacterial genera which were significantly correlated with the surface sediment samples. These included *Altererythrobacter, Alteromonas, Aquisphaera, Blastopirellula, Erythrobacter, Gaetbulibacter, Halobacteriovorax, Henriciella, Maricaulis, Marinobacter, Marinomonas, Marivirga, Marivita, Paraglaciecola, Parvularcula, Phycisphaera, Planctomicrobium, Pseudoalteromonas, Robiginitalea, Salinisphaera,* and *Sphingobium*. A single archaeal OTU was significantly correlated with surface samples, a *Halarchaeum.*

As microbial communities varied more by depth than hydration, there were many more significantly differing taxa by depth in a Metastats analysis. Therefore, we summarized depth differences with an alpha cutoff of 0.01 and hydration differences by a 0.05 cutoff. The analysis separated surface from subsurface sediments by generally the same taxa as the Spearman correlation (Table 2), emphasizing the disproportionate abundance of those marine and halophilic taxa in the surface rather than the subsurface. A separate Metastats analysis (α = 0.05) showed that there were 11 bacterial taxa that were significantly more abundant in the surface layer under the hydrated state specifically (Table 3). Among these taxa were members of the families Planctomycetaceae, Flavobacteriaceae, Bdellovibrionaceae, Oceanospirillaceae, Rhodospirillales, Flammeovirgaceae, and Cytophagaceae. No other pairwise comparison of hydration and depth showed significantly correlated bacterial taxa. There were 28 archaeal OTUs that were significantly different in relative abundance between the surface and subsurface, but only one, a Halobacteriaceae, differed significantly between the hydration states, being more abundant in the hydrated samples (Table 4). None of the archaeal OTUs differed significantly in abundance between the pairwise combinations of layer and hydration. 

**Figure 4:**
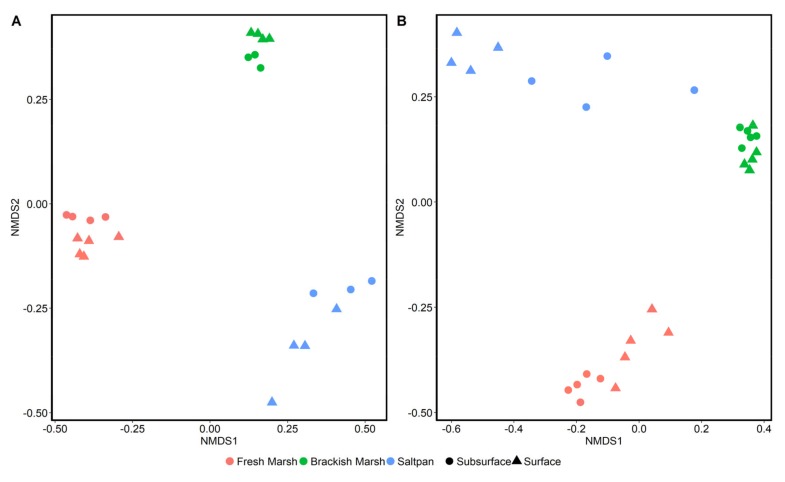
NMDS ordination of bacterial (**A**) and archaeal (**B**) communities in wetland sediment, collected from Grand Bay National Estuarine Research Reserve in southeast Mississippi, USA. Wetland salinity class is represented by color and soil level represented by shape. Ordinations are based on Bray–Curtis dissimilarity matrices derived from 16S rRNA gene sequence data.

The microbial communities from the saltpan site were compared against those in sediment at freshwater and brackish marsh sites also located at GBNERR. A Bray–Curtis distance matrix and NMDS ordination (Figure 4) clearly separated sequences into three distinct clusters by wetland type, as microbial sequences at each of the three wetland types differed significantly in archaeal and bacterial composition (*p* < 0.001 for both). As with the saltpan site alone, the effect of soil depth was also a significant factor across all three wetland classes for both the bacterial (*p* = 0.029; PERMANOVA) and archaeal communities (*p* = 0.037).

The Piphillin metagenomic inference tool was used for mapping 16S rRNA amplicons to predicted genomes and the MetaCyc metabolic pathway database was used for grouping pathways into differentially expressed functional groups. This pipeline predicted significant differences between the functionality of the saltpan in the desiccated and hydrated state. However, there were no significant differences observed between the genomes of the surface and 30 cm deep samples, and no differences were resolved between the hydration states of the individual layers. A Mantel’s test showed a weak correlation between the distance matrices of the taxonomic and predicted metagenomic sequences of the saltpan samples (Mantel’s *r*= 0.34, *p* = 0.087), indicating low confidence in the functional predictions at that site alone. The Piphillin/MetaCyc pipeline yielded stronger results in predicting the genomes across all three wetland types (saltpan, freshwater marsh, brackish marsh), showing multiple significant differences in functional group abundances between the three wetland classes. A Mantel’s test showed weak but significant correlation between the taxonomic and genomic distance matrices across the three sites (*r* = 0.24, *p* = 0.004). 

**Figure 5:**
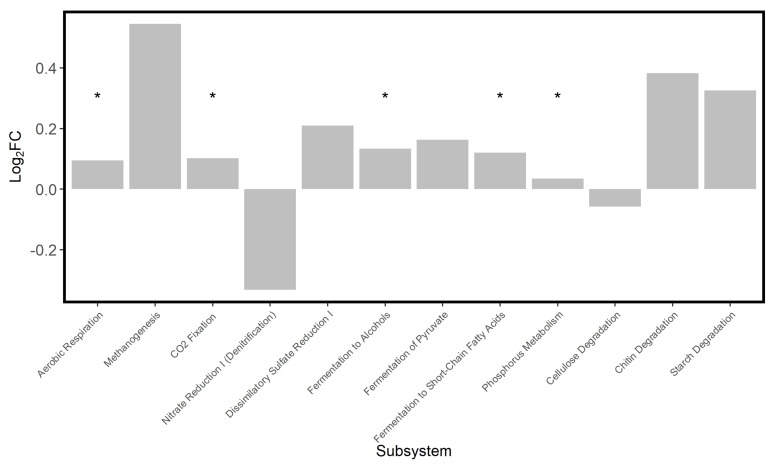
Differential abundance of MetaCyc subsystems in microbial communities in saltpan wetland sediment when hydrated compared to desiccated. Log_2_FC, fold change, represents the shift from the desiccated to the hydrated state and those functional groups which were represented by more amplicons following the flood event show positive change and those which had fewer amplicons show negative change. MetaCyc enrichment analysis determined which functional groups showed a significant change (*), based on the raw number of daughter pathways which changed in amplicon number.

**Figure 6:**
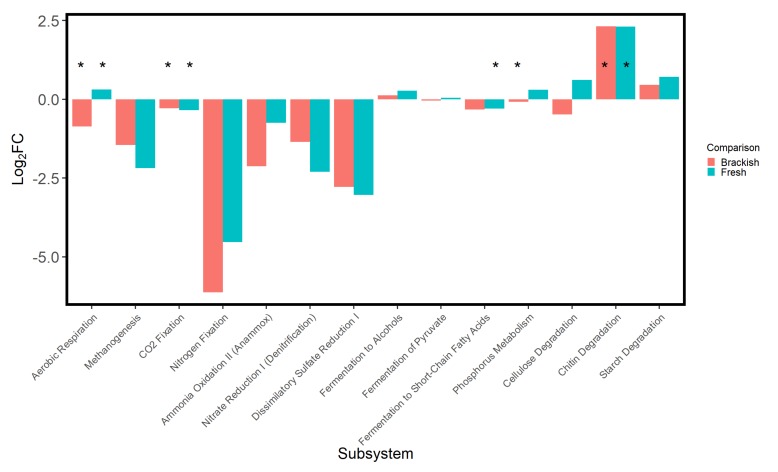
Differential abundance of MetaCyc subsystems in microbial communities in brackish and tidal fresh wetlands as compared to a saltpan wetland in the same area of Grand Bay National Estuarine Research Reserve in southeast Mississippi, USA. Log_2_FC was used as a measure, of which functional groups had more or fewer pathways expressed when comparing each tidal wetland to the saltpan based on differences in the taxa present. Fold change represents the difference between the saltpan amplicon counts and the amplicons counts of the brackish (red) and fresh (blue) tidal wetlands. Those functional groups which were represented by more amplicons in either the brackish or fresh wetland show positive change and those which had more amplicons in the saltpan sediment show negative change. MetaCyc enrichment analysis determined which functional groups showed a significant difference (*), based on the raw number of daughter pathways which differed in amplicon number.

The hydrated saltpan samples showed increases of ~0.3–0.5 log_2_FC in pathways associated with chitin and starch degradation as well as those involved in methane production (Figure 5), indicating a ~23%–41% upregulation of those metabolic functions. Most subsystems showed upregulation, with the exceptions of a marginal decrease in cellulose degradation pathways and a −0.33 log_2_FC decrease in denitrification pathways. Enrichment analysis, which quantifies functional change by the number of pathways which differed, showed significantly more pathways enriched for aerobic respiration, CO_2_ fixation, fermentation, and phosphorus metabolism (*p* < 0.05). When compared with saltpan samples, samples from both the adjacent brackish and fresh marsh showed >2-fold fewer pathways associated with methanogenesis, denitrification, and sulfate reduction and ~30-fold fewer pathways involved in nitrogen fixation (Figure 6). Both tidal wetland types also showed a significant and ~4-fold enrichment in chitin degradation. 

## 4. Discussion

This study represents, to our knowledge, the first description of the sediment microbiome of a saltpan wetland. Our findings refute previous descriptions of saltpans and other tidal flats as ecologically unproductive, their spread in area having been likened to desertification [2,37]. Sea level models have projected that saltpans and tidal flats may expand greatly in area in response to vegetation loss to saltwater intrusion [9,38]. Within the GBNERR itself, models project an expansion of transitional salt marsh and tidal flats, wetlands that readily allow for saltpan formation [2]. The replacement of tidal fresh, brackish, and salt marsh by upland salt marshes, saltpans, and other tidal flats underscores the importance of understanding the microbial community of these habitats, which are the primary drivers of carbon storage and nutrient cycling in those ecosystems [10,11,12].

We found that, at the phylum level, the saltpan bacterial community was dominated by Proteobacteria, with Bacteroidetes and Planctomycetes dominating the remaining fraction. This finding is in agreement with commonly found patterns in both salt marsh [39,40] and tidal flat habitats [41]. Of the sequences classified at the genus level, *Pseudomonas* dominated and pseudomonads, as well as *Flavobacterium,* have been reported in coastal beach sediment, along with other genera characterized by salt tolerance and facultative anaerobic metabolisms [42,43]. These taxa were associated with high- and ebb-tide samples [43], when sediments would be inundated and anoxic, relying on alternative electron acceptors. Our site was in a similar state during the October sampling; inundated but ebbing one month after a storm tide. We did not observe a significant difference in the relative abundance of sequences classified as *Pseudomonas* between hydration states, but Flavobacteriaceae were solely detected in the hydrated surface samples and were strongly correlated with that state according to the Metastats calculation. This could indicate that a niche for Flavobacteriaceae is present in the inundated sediment, but not when desiccated.

As predicted, recovered sequences, species richness, and diversity were all much lower than in studies of other, more moderate wetland types. One study of the Pearl River estuary in subtropical China [44] observed Shannon diversity between 10.84 and 11.58, while we observed an average of 3.94. Likewise, they observed archaeal Shannon values between 7.51 and 7.93, while we observed ~2.38. A study of a brackish lagoon in the Bay of Bengal [45] reported an average inverse Simpson score of 169 and ~13,000 sequences per sample, compared to 1305 sequences and an average inverse Simpson score of 42 for our data. While some of these differences could be attributable to different sizes of datasets, the low observed richness and diversity within saltpan sediments likely reflect true, highly restrictive conditions. Enforcing this conclusion is that inverse Simpson diversity averaged 368 and 288 at the brackish and freshwater marshes, respectively, sampled within close proximity to the saltpan, versus 42 in sediments from the saltpan itself. This was true despite greater coverage in the saltpan samples than in the other wetland types. 

Many of the prominent bacterial taxa that were observed in saltpan sediments are common seawater and coastal marine microorganisms. Generally, the surface sediment was dominated by common marine taxa [46,47,48,49,50,51,52,53,54,55,56,57,58,59,60,61,62] and several others which have been previously isolated from tidal flats [63,64,65], salt evaporates [66], and other saline environments [67,68,69]. Based on relative abundance, many of these taxa are likely transient and occur with flood or storm tides. The subsurface sediment had a larger community of likely anaerobes and facultative taxa, including *Aeromonas* and *Bacteroides* [70,71], as well as the endospore formers, *Bacillus* and *Paenibacillus* [72]. The hydrated samples were separated from the desiccated state by previously identified marine sediment bacteria [73,74,75,76], as well as the genera *Mycobacterium,* commonly known as a pathogen [77], *Luteolibacter*, previously identified in Arctic soil [78], and *Methyloceanibacter*, isolated from near a hydrothermal vent [79]. Among the archaeal sequences, the surface samples were characterized by an abundance of Halobacteriaceae and Haloferacaceae, both extreme halophiles [80,81]. The subsurface sediment was significantly correlated with Nitrososphaera, an ammonia oxidizer, and Methanomassiliicoccaceae, an anaerobic methanogen [82,83].

It is possible that the unexpected patterns of saltpan community structure, influenced more by depth than hydration, could be due to the tradeoffs between niche and stochastic processes. pH, organic matter, and particle sizes were most similar between sediment layers, regardless of hydration. Perhaps most importantly, salinity was reduced by ~10.9 PSU in the surface layer following flood tide, while it was reduced by only ~1.3 PSU in the root zone layer. This confirmed our only correct prediction: that sediment compaction would prevent the disturbance effect of hydration from reaching the lower sediment layer. Within-layer partitioning of these abiotic factors likely drives the observed differences in community composition by soil depth rather than hydration [84]. Alternatively, soil depth differences could still be driven by the flood event, but through stochastic processes [85]. The Metastats analysis, based on relative abundance of OTUs, showed that the most differentially present taxa between sediment levels were marine genera. Their arrival through the storm tide represents a random dispersal event and we predict that if this system were followed through to complete desiccation, these taxa would be largely absent. The observed transition from a halophilic and desiccation-tolerant surface community to one dominated by marine taxa would then result from both niche and stochastic processes. The abundance of such halophiles as *Halarchaeum,* Rhodothermaceae, and *Truepera* [86,87,88] did not differ by depth or hydration and are likely able to exploit niches unaffected by differences in soil moisture. They would only become less abundant relative to marine taxa due to random flood immigration events. Finally, such halophiles as *Salinisphaera, Marivita, Alteromonas, Marinobacter, Halobacteriaceae,* and *Haloferacaceae* [66,67,68,69,80,81] were restricted to the surface alone and their relative abundance may be due to either effect, likely receiving contributions from both deterministic and random processes.

Rather than having a disturbance effect, the inferred metagenomes of the saltpan samples following the hydration event were shown to be moderately enriched. Of the 12 energy, degradation, and assimilation pathways examined, 10 were enriched in the hydrated rather than the desiccated state. This included promotion of aerobic respiration, anaerobic metabolisms such as sulfate reduction, fermentation, and the degradation of carbon compounds chitin and starch. This could be comparable to stimulation of activity in desert soils following rain events [89]. It should be noted, though, that the stimulation of desert soil was independent of significant changes to community composition, while our inference is based on correlations to compositional change. It was unexpected that the inferred metagenomes of the saltpan showed significant differences between dry and flooded states but not with depth, despite the fact that composition was structured by depth alone. This underscores the need for further study of the functional processes in saltpans and similar unvegetated wetlands. 

Inferred genomes were more informative and significantly correlated to taxonomy across the three wetland types. Microbial communities differed in both composition and inferred activity between the saltpan and adjacent brackish and freshwater marshes. This is interesting, given that each wetland type receives the same nutrient inputs from tides and river deposition and underscores the strong selective pressure that salinity has on wetland bacterial communities [90,91,92]. It should be noted, though, that none of the saltpan samples observed were above ocean salinity at the time of sampling and during the hydrated state, salinities were comparable to brackish marsh. While the site is known to exhibit hypersaline conditions periodically [6], this implies that other physico-chemical differences contribute significantly to compositional differences between the wetland types. 

It is projected that modern tidal fresh marsh area will be lost gradually to brackish marsh and ultimately to transitional salt marsh and tidal flats [7], both environments suitable to the formation of saltpans. Thus, it is important to better characterize the microbial community composition and activity of each of these habitats. In sharp contrast to the suggestion that saltpans are unproductive [2] or that the conversion of tidal marsh to saltpan is comparable to desertification [37], the inferred metagenomes suggested that the majority of the energy, degradation, and assimilation pathways observed were actually repressed in brackish and fresh marsh, as compared to saltpan sediments. While significant based on the number of pathway changes, differences in pathways associated with aerobic respiration, carbon fixation, and phosphorus metabolism were only moderate in magnitude, but predicted methanogenesis, nitrogen fixation, anammox, denitrification, and sulfate reduction where much greater in saltpan sediments than in brackish or fresh sediment. Predicted capability for methanogenesis was higher in freshwater than brackish marsh, in agreement with previous studies [93,94], but was highest in saltpan sediments. Methanogenesis is frequently observed in hypersaline environments [95] and the potential for the process here is attributable to the prevalence of sequences identified as methanogenic Euryarchaeota [96]. Increased sulfate reduction in response to saltwater intrusion is a common phenomenon [97] and may help to explain the high potential for nitrogen fixation observed in saltpan sediments, as nitrogen fixation can be coupled to sulfate reduction [98,99,100]. Some Euryarchaeota are also able to fix nitrogen and are beginning to be recognized for their contribution to organic nitrogen supply in soils [101]. These unexpected differences in potential energy and nutrient flows between saltpans and brackish and fresh marsh underscore the inferential nature of the current methods and imply that further functional as well as taxonomic investigation of saltpan sediments are warranted.

## 5. Conclusions

This study shows that the microbial communities of saltpans are primarily constrained by sediment depth, with surface sediments differing significantly in composition from those deeper in the sediment. Saltpan microbial communities were similar in composition in desiccated and hydrated states, with the abundance of many marine bacterial taxa in the hydrated state likely attributable to transient marine taxa that are unable to persist in the hypersaline and arid long-term conditions of the saltpan. The saltpan microbiome hosts multiple extremophilic taxa and an inferred metagenome revealed surprisingly high rates of energetic and nutrient cycling pathways, even when compared with adjacent brackish and fresh marsh. These differences correlated with large taxonomic differences between saltpan, fresh, and brackish marsh, even across small spatial scales. With saltwater intrusion projected to replace fresh and brackish marsh habitat with altered conditions suitable to saltpan expansion, it will be critical to consider the saltpan wetland microbiome when budgeting the provision of ecosystem services in the future. 

## Figures and Tables

**Table 1:** Sediment physico-chemical characteristics of a saltpan wetland along the Mississippi Gulf Coast. Salinity (PSU) and pH were calculated by hydrating sediment in a 1:5 dilution in the lab and measured using a YSI probe and pH electrode. Percent organic matter (OM%) was calculated by loss on ignition. Sand silt and clay fractions were calculated by sieving the 1:5 dilution through a 0.053 mm mesh (sand) and separating silt from clay by settling for 4 h. The dry weight of each fraction was divided by the original dry sample mass to derive the percentage composition of each. Salinity, pH, and OM% were performed in triplicate, while particle sizes were determined from a pooling of the triplicates.

Parameter	Desiccated Surface	Hydrated Surface	Desiccated Subsurface	Hydrated Subsurface
Salinity (PSU)	25.6 ± 0.13	14.7 ± 0.06	14.9 ± 0.03	13.6 ± 0.06
pH	5.91 ± 0.02	6.31 ± 0.11	6.33 ± 0.06	6.82 ± 0.04
OM (%)	5.53 ± 0.35	4.73 ± 0.03	4.08 ± 0.23	3.43 ± 0.22
%Sand	67.9	62.8	43.5	36.8
%Silt	28.7	35.1	49.8	56.9
%Clay	3.4	2.0	6.7	6.3

**Table 2:** Bacterial taxa that were significantly more abundant in 16S rRNA sequence data from saltpan wetland sediments in either a desiccated or hydrated state, or in the surface or 30 cm deep subsurface. Community composition differed significantly by depth but not hydration, so α = 0.01 was used for summarizing differences by depth and α = 0.05 for summarizing differences by hydration state. Only those OTUs which had a significant *p*-value and were identified at the genus level are shown.

Surface			Subsurface		
OTU	Identity	*p*-value	OTU	Identity	*p*-value
15	*Truepera*	0.001	3	*Pseudomonas*	0.008
22	*Salinisphaera*	0.004	7	*Providencia*	0.004
43	*Marivita*	0.007	35	*Aeromonas*	0.007
91	*Alteromonas*	0.002	54	*Stenotrophomonas*	0.005
106	*Blastopirellula*	0.003	63	*Alcaligenes*	0.008
131	*Erythrobacter*	0.010	439	*Bacteroides*	0.002
138	*Halobacteriovorax*	0.007	444	*Bacillus*	0.010
170	*Henriciella*	0.002	674	*Paenibacillus*	0.007
171	*Aquisphaera*	0.005	741	*Bacteroides*	0.005
221	*Robiginitalea*	0.001			
353	*Geodermatophilus*	0.003			
367	*Pseudoalteromonas*	0.009			
384	*Parvularcula*	0.003			
548	*Vampirovibrio*	0.006			
583	*Parvularcula*	0.007			
**Desiccated**			**Hydrated**		
OTU	Identity	*p*-value	OTU	Identity	*p*-value
860	*Flavobacterium*	0.046	319	*Muricauda*	0.048
			341	*Vampirovibrio*	0.046
			346	*Mycobacterium*	0.020
			389	*Luteolibacter*	0.021
			404	*Marinobacterium*	0.041
			435	*Aquisphaera*	0.025
			529	*Methyloceanibacter*	0.020
			590	*Marivirga*	0.038
			688	*Ilumatobacter*	0.040

**Table 3:** Bacterial taxa that were significantly more abundant in 16S rRNA sequence data from a saltpan wetland in hydrated surface sediments. The means in either the hydration state or depth are the relative abundance outputs of a Metastats calculation. The lowest level taxonomy of each classified OTU is presented. Many of the taxa are potential marine transients, while others are possible resident halophiles.

OTU	Identity	Mean (Hydrated)	Mean (Desiccated)	*p*-value	Mean (Surface)	Mean (Subsurface)	*p*-value
102	Planctomycetaceae	1.61 × 10^−3^	3.23 × 10^−4^	0.031	1.67 × 10^−3^	2.63 × 10^−4^	0.017
319	Flavobacteriaceae	3.18 × 10^−4^	0.00	0.048	3.18 × 10^−4^	0.00	0.047
341	Bdellovibrionaceae	3.66 × 10^−4^	0.00	0.046	3.66 × 10^−4^	0.00	0.046
342	Alphaproteobacteria (c)	3.04 × 10^−4^	0.00	0.044	3.04 × 10^−4^	0.00	0.044
403	Alphaproteobacteria (c)	2.05 × 10^−4^	4.90 × 10^−5^	0.035	2.04 × 10^−4^	5.00 × 10^−5^	0.043
404	Oceanospirillaceae	2.57 × 10^−4^	0.00	0.041	2.57 × 10^−4^	0.00	0.041
470	Unclassified	1.62 × 10^−4^	0.00	0.049	1.62 × 10^−4^	0.00	0.049
571	Rhodospirillales	1.42 × 10^−4^	0.00	0.04	1.42 × 10^−4^	0.00	0.04
590	Flammeovirgaceae	1.35 × 10^−4^	0.00	0.038	1.35 × 10^−4^	0.00	0.036
614	Cytophagaceae	1.35 × 10^−4^	0.00	0.038	1.35 × 10^−4^	0.00	0.036
668	Flavobacteriaceae	1.22 × 10^−4^	0.00	0.046	1.22 × 10^−4^	0.00	0.046

**Table 4:** Archaeal taxa that were significantly more abundant in 16S rRNA sequence data from saltpan wetland sediments in either a desiccated or hydrated state, or in the surface or 30 cm deep subsurface. Only those OTUs which had a significant *p*-value and were identified at the family level are included. There were no archaeal OTUs that were significantly more correlated with the desiccated state than the hydrated state.

Surface			Subsurface		
OTU	Identity	*p*-value	OTU	Identity	*p*-value
2	Halobacteriaceae	0.014	11	Nitrososphaera	0.009
4	Halobacteriaceae	0.023	42	Nitrososphaera	0.008
19	Halobacteriaceae	0.035	60	Methanomassiliicoccaceae	0.028
22	Haloferacaceae	0.033			
23	Halobacteriaceae	0.030			
25	Halobacteriaceae	0.022			
27	Haloferacaceae	0.036			
45	Halobacteriaceae	0.043			
**Hydrated**					
OTU	Identity	*p*-value			
1	Halobacteriaceae	0.023			
